# ENaC Biomarker Detection in Platelets Using a Lateral Flow Immunoassay: A Clinical Validation Study

**DOI:** 10.3390/bios15070399

**Published:** 2025-06-20

**Authors:** Giosvany Martínez-Boloña, Ivette Martínez-Vieyra, M. B. de la Mora, Marco Antonio Fuentes-García, César Reyes-López, Doris Cerecedo

**Affiliations:** 1 Laboratorio de Hematobiología, Escuela Nacional de Medicina y Homeopatía, Instituto Politécnico Nacional, Ciudad de México 07700, Mexico; gmartinezb2302@alumno.ipn.mx (G.M.-B.); iamartinez@ipn.mx (I.M.-V.); 2 Instituto de Ciencias Aplicadas y Tecnología UNAM, Circuito Ext. S/N, Cd. Universitaria, Ciudad de México 04510, Mexico; maria.delamora@icat.unam.mx; 3 Unidad de Investigación en Biomedicina y Oncología Genómica, Hospital de Gineco Pediatría 3A, OOAD Norte, Instituto Mexicano del Seguro Social, Mexico City 07760, Mexico; marco.fuentesg@imss.gob.mx; 4 Laboratorio de Bioquímica Estructural, Escuela Nacional de Medicina y Homeopatía, Instituto Politécnico Nacional, Mexico City 07230, Mexico; careyes@ipn.mx

**Keywords:** high blood pressure, platelets, epithelial sodium channels, lateral flow immunoassays, gold nanoparticles, diagnostic, sensitivity, specificity

## Abstract

Arterial hypertension (HTN) is a growing global health concern, with limited tools available for early detection. Previous studies identified the overexpression of the epithelial sodium channel (ENaC) as a potential biomarker for HTN. In this work, we optimized and clinically validated a lateral flow immunoassay (LFIA) using gold nanoparticles (AuNPs) functionalized with anti-ENaC antibodies. The test strips were prepared with 10 µL of each component and performed in a 9-point herringbone format. For validation, a double-blind study was conducted using platelet lysates from 200 individuals, classified based on real-time blood pressure measurements. ENaC expression was assessed via both LFIA and Western blotting, which served as the reference method. Receiver operating characteristic (ROC) analysis yielded an AUC of 0.7314 for LFIA and 0.6491 for the Western blot, with LFIA demonstrating higher sensitivity (76.24%) and comparable specificity (61.54%) compared to the Western blot (68.31% and 60.34%, respectively). These results support LFIA as a practical, rapid, and moderately accurate tool for screening ENaC levels and identifying individuals at risk of hypertension.

## 1. Introduction

Arterial hypertension (HTN) is a chronic condition characterized by elevated pressure exerted by the heart as it pumps blood into the arteries to maintain systemic circulation. HTN is one of the leading causes of death globally and a major risk factor for cardiovascular, cerebrovascular, and renal diseases. Approximately 95% of hypertension cases are classified as “primary” or “essential”, meaning that they lack a clearly identifiable cause. Nonetheless, several factors are associated with its development, including advancing age, a high sodium intake, diets rich in saturated fats, smoking, physical inactivity, and chronic conditions such as obesity, dyslipidemia, and diabetes [1].

Despite these known associations, a large number of individuals with hypertension remain asymptomatic, making early detection particularly difficult [2].

When left undiagnosed or poorly managed, hypertension gradually worsens, substantially increasing the risk of serious complications such as stroke, heart failure, myocardial infarction, aneurysm, atherosclerosis, vision loss, and kidney damage. Therefore, the timely diagnosis and effective management of hypertension are essential for reducing morbidity and mortality, as the severity of complications correlates directly with both the level and duration of elevated blood pressure [3,4].

Blood pressure measurement remains the primary tool for diagnosing hypertension. For many years, considerable debate surrounded the threshold values associated with cardiovascular risk and the formal diagnosis of the disease. A global consensus was eventually reached, defining hypertension in adults as consistent blood pressure readings exceeding 140/90 mmHg [5].

While the clinical diagnosis is usually straightforward when blood pressure readings are clearly and consistently elevated over 2–3 weeks in various settings and times of day, challenges arise when readings fluctuate around the 140/90 mmHg threshold—sometimes exceeding it and at other times falling below. Moreover, blood pressure naturally varies between individuals and is influenced by complex interactions among cardiovascular regulatory mechanisms, physical activity, environmental factors, and circadian rhythms [6].

Due to the importance of the accurate and early diagnosis of hypertension, various research groups have developed innovative diagnostic tools [7]. These range from the evaluation of physiological signals and the application of machine learning algorithms to analyze clinical data and photoplethysmography signals to the identification of biomarkers that aid in predicting the risk of hypertension [8].

The epithelial sodium channel (ENaC) is a heterotrimeric channel composed of three homologous subunits (α, β, and γ) that are expressed in both epithelial and non-epithelial cells across various tissues and organs [9], playing a crucial role in regulating fluid volume, sodium homeostasis, and, consequently, blood pressure [10]. Therefore, the ENaC has been proposed as a potential biomarker for hypertension [11]. Several approaches have been developed to detect the ENaC in biological samples such as urine and blood, ranging from electrochemical immunosensors [12,13] to immunoassay-based platforms.

Recently, our research group reported a correlation between ENaC activity in platelets and arterial hypertension. To support this finding, we employed a bioconjugate consisting of gold nanoparticles (AuNPs) functionalized with an anti-ENaC antibody as a complementary diagnostic tool. A subsequent double-blind clinical study in an open population (*n* = 167) further demonstrated the potential of this biomarker for hypertension screening [14].

Biomarker-based diagnostics not only aid in the detection of hypertension but also provide valuable insights into the physiological alterations associated with the disease. A range of biomarkers, including reactive oxygen species (ROS), cortisol, aldosterone, leptin, cyclophilin A, C-reactive protein, ghrelin, resistin, chemerin, brain natriuretic peptide, and cardiac troponin T, has been identified using enzyme-linked immunosorbent assays (ELISAs) [15].

Among current diagnostic platforms, the lateral flow immunoassay (LFIA) has emerged as a promising tool due to its low cost, rapid turnaround time, and operational simplicity, especially for the early detection of disease biomarkers, even when present at low concentrations [16].

To enhance the performance and practicality of LFIA diagnostics, various metal nanoparticles (NPs)—including gold nanoparticles (AuNPs), carbon NPs, quantum dots (QDs), and upconversion nanoparticles (UCNPs)—have been integrated to leverage their unique optical properties, as recently reviewed [17].

Based on these findings, we developed a prototype lateral flow immunoassay. Spherical gold nanoparticles (AuNPs) were synthesized using the sodium citrate reduction method and subsequently conjugated with anti-ENaC antibodies to form stable bioconjugates optimized for immunochromatographic applications. Clinical validation was conducted using platelet lysates from 200 individuals attending the Unidad de Medicina Familiar No. 44 of the Instituto Mexicano del Seguro Social in Mexico City, regardless of their blood pressure status or history of hypertension. The assay’s sensitivity and specificity were evaluated against Western blotting, the current gold standard for ENaC protein detection.

The lateral flow test effectively confirmed hypertension diagnoses and identified a considerable number of previously undiagnosed cases, demonstrating its potential as a screening tool. Although these results represent a promising step toward the development of a point-of-care diagnostic assay for hypertension, further optimization is required to enhance signal intensity and improve test line visibility.

## 2. Materials and Methods

**Figure 1:**
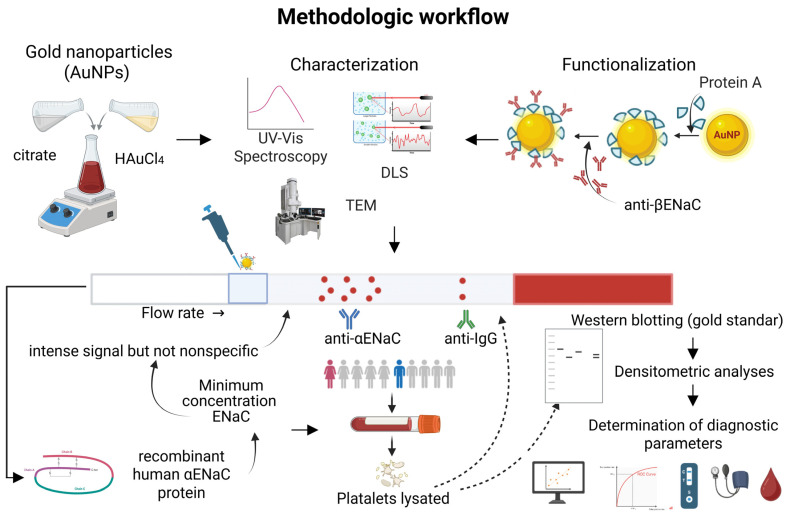
Experimental workflow diagram. Schematic representation of the experimental procedures followed in this study, including sample collection, nanoparticle synthesis and functionalization, assay development, and data analysis. Each step illustrates the logical progression and methodological integration throughout the study. Created by Boirender.com.

The overall experimental design and methodological approach described throughout this study are summarized in Figure 1, which provides a visual representation of each major step—from nanoparticle synthesis and functionalization to assay development, clinical sample analysis, and data interpretation. 

### 2.1. Gold Nanoparticle Synthesis

Gold nanoparticles (AuNPs) were synthesized using the Turkevich method (sodium citrate reduction) [18]. Briefly, 20 mL of a 25 mM tetrachloroauric acid (HAuCl_4_) solution (1%, Cat. No. 520918; Sigma-Aldrich, St. Louis, MO, USA) was added to a 50 mL glass beaker. A separate 1% sodium citrate solution (Cat. No. 0101-1KG; Amresco, Solon, OH, USA) was prepared in parallel. The HAuCl_4_ solution was heated to boiling on a magnetic stirring hotplate (Thermo Scientific), after which 1 mL of the sodium citrate solution was added. Boiling and continuous stirring were maintained until the solution turned deep red, indicating AuNP formation. The mixture was then cooled to room temperature, transferred to glass vials, and stored at 4 °C. Prior to its use, excess sodium citrate was removed by washing the AuNP solution with double-distilled water (500 μL per 1 mL of the AuNP solution), followed by centrifugation at 3500 rpm for 40 min to concentrate the nanoparticles. In addition to synthesized AuNPs, commercially available AuNPs of different sizes were evaluated to determine the optimal particle diameter for the assay: 40 nm AuNPs (Cat. No. 741981; Sigma-Aldrich, St. Louis, MO, USA) and 150 nm AuNPs (Cat. No. GSXR150-10M; NanoComposix, San Diego, CA, USA).

### 2.2. UV-Vis Absorption Spectroscopy

A sample of gold nanoparticles (AuNPs) was analyzed using a UV–Vis spectrophotometer over a wavelength range of 200 to 800 nm. The absorbance spectrum was recorded, and the resulting data were plotted to evaluate the optical properties of the AuNPs. The size and homogeneity of the synthesized nanoparticles were estimated based on the position and shape of the surface plasmon resonance (SPR) peak, which reflects particle diameter and dispersion characteristics.

### 2.3. Dynamic Light Scattering

The gold nanoparticle (AuNP) sample was prepared by dispersing the particles in a stabilization buffer composed of 0.01 M phosphate-buffered saline (PBS), 0.5% sucrose, 2% bovine serum albumin (BSA), and 0.1% Tween 20. Dynamic light scattering (DLS) measurements were conducted using a Zetasizer Nano Series instrument to determine the hydrodynamic diameter of the AuNPs. The DLS data were used to calculate the size distribution and polydispersity index (PDI), providing an assessment of nanoparticle dispersion and homogeneity.

### 2.4. Transmission Electron Microscopy

The morphology and size of the synthesized gold nanoparticles (AuNPs) were examined using transmission electron microscopy (TEM). A 2 μL aliquot of the nanoparticle suspension was deposited onto a carbon-coated copper grid and air-dried at room temperature. Images were acquired at various magnifications to ensure detailed structural characterization. Focus, contrast, and brightness were adjusted to enhance image clarity. To ensure representativeness, images were captured from multiple, randomly selected areas of the grid, minimizing sampling bias. The average particle diameter was determined by measuring the full width of individual nanoparticles from the TEM images using the ImageJ 1.53 k software.

### 2.5. Functionalization of AuNPs with Protein A and the Anti-βENaC Antibody

To optimize the lateral flow immunoassay (LFIA) platform, gold nanoparticles (AuNPs) were functionalized with Protein A to facilitate antibody binding. Following previously established protocols [19], the minimum concentration of Protein A required to stabilize AuNPs was first determined. Various concentrations of Protein A (0.2, 0.3, 0.5, 1.0, 3.0, 6.0, and 9.0 μg/mL; Cat. No. P6031-5MG, Sigma-Aldrich, St. Louis, MO, USA) were evaluated.

To identify the optimal concentration for AuNP stabilization, 30–90 μL of Protein A (100 μg/mL) was added to 1 mL of the AuNP solution. After a 10 min incubation period at room temperature, 1 mL of 10% sodium chloride (NaCl; Cat. No. S9625, Sigma-Aldrich) was added to induce nanoparticle aggregation. A 200 μL aliquot of each reaction mixture was transferred into a 96-well plate (Cat. No. 3855, Thermo Scientific, Rochester, NY, USA), and changes in solution color (from red to blue) and absorbance were recorded. The minimum Protein A concentration that prevented color change and maintained a stable absorbance spectrum was selected as the optimal condition for coating.

Following optimization, AuNPs were functionalized by incubating with the selected concentration of Protein A for 1 h at room temperature. The conjugated nanoparticles were then washed with 0.01 M phosphate-buffered saline (PBS) containing 0.1% bovine serum albumin (BSA; Cat. No. A8022-10G, Sigma-Aldrich) to remove unbound Protein A. Centrifugation was performed at 3500 rpm for 40 min. A final blocking step with 0.01 M PBS containing 3% BSA was carried out for 1 h to minimize nonspecific binding, followed by another centrifugation under the same conditions. The functionalized AuNPs were stored at 4 °C until further use.

Prior to this, three resuspension buffers for the functionalized AuNPs were evaluated:

Buffer 1: 0.01 M PBS, 5% sucrose, 1% BSA, and 0.5% Tween-20;

Buffer 2: 0.01 M PBS, 10% sucrose, 1% BSA, and 0.1% Tween-20;

Buffer 3: 0.01 M PBS, 0.5% sucrose, 2% BSA, and 0.1% Tween-20.

The functionalized nanoparticles were resuspended in Buffer 3 because it showed the best colloidal stability, as assessed by UV-Vis spectroscopy, dynamic light scattering (DLS), and zeta potential measurements. Based on these characterizations, the Protein A concentration was first reduced to 0.5 μg/mL and later optimized to 0.2 μg/mL to minimize nonspecific interactions in the LFIA strips.

Following Protein A optimization, the AuNP–Protein A complex was incubated with a monoclonal anti-β-ENaC antibody (Cat. No. βENaC (E-10): sc-48428; Santa Cruz Biotechnology, Inc., Dallas, TX, USA) at an optimal concentration of 0.5 μg/mL for 1 h at room temperature. The antibody concentration was established through preliminary assays to ensure effective binding and assay performance. After incubation, the conjugated complex was washed by centrifugation and resuspended in the selected stabilization buffer.

To confirm successful antibody conjugation, the functionalized AuNP–Protein A–anti-β-ENaC complex was characterized using a combination of analytical techniques. UV–visible (UV-Vis) spectroscopy was employed to assess surface plasmon resonance (SPR) shifts indicative of protein binding. Dynamic light scattering (DLS) and zeta potential measurements were used to evaluate changes in hydrodynamic size, surface charge, and colloidal stability. Additionally, transmission electron microscopy (TEM) was performed to visually inspect particle morphology and assess the uniformity of conjugation. These analyses collectively confirmed effective antibody binding and guided the selection of the optimal nanoparticle source (synthesized vs. commercial) and size for use in the final lateral flow immunoassay (LFIA).

### 2.6. Optimization of Running Buffers and Membrane Flow Conditions

To ensure optimal capillary flow and consistent performance in the lateral flow immunoassay (LFIA), various running buffers were tested for compatibility with the assay system. The evaluated buffers included (1) a borate buffer, pH 7; (2) Buffer 2 (1% BSA, 1% sucrose, 0.05 M NaCl, 0.05 M L-arginine, 0.02% PEG, 0.5% polyvinyl alcohol, and 0.2% Tween-20); (3) a Tris-EDTA buffer; and (4) a PBS-based buffer (0.01 M PBS, 1% Tween-20, and 1% BSA). Each buffer was applied to the sample pad at a volume of 85 μL, which was identified as the minimum volume required for the complete migration of the sample to the absorbent pad.

For these evaluations, LFIA strips assembled with 50 μm nitrocellulose membranes (Cat. No. HF18002XSS, Hi-Flow™ Plus, MERCK, Macquarie Park, Australia), exhibiting a flow rate of 180 s/4 cm, were used. Migration was monitored for 15–20 min until the signal reached the excess (absorbent) pad. In addition to buffer composition, membrane characteristics were also assessed for their impact on flow dynamics. Two types of membranes were compared: (1) 50 μm, 180 s/4 cm (HF18002XSS) and (2) 100 μm, 75 s/4 cm (HFC07504XSS).

The combined evaluation of buffer formulations and membrane properties allowed for the selection of conditions that supported reproducible flow, enhanced signal clarity, and overall assay reliability in the LFIA system.

### 2.7. Components of the Immunological Reaction

To construct the test and control zones of the lateral flow immunoassay (LFIA) strip, specific antibodies were immobilized onto the nitrocellulose membrane. For the test line, an anti-αENaC antibody (Cat. No. PA1-920A; Invitrogen, Rockford, IL, USA), which specifically recognizes amino acids 20–44 of the intracellular domain of the human α-subunit of the ENaC, was utilized. Initially applied at the manufacturer-recommended concentration of 1 mg/mL, the antibody was subsequently diluted to a final working concentration of 0.5 mg/mL in a blocking solution containing 1% non-fat dry milk and Protein A. This adjustment was made to reduce nonspecific interactions and improve the specificity of signal development at the test line.

For the control line, an anti-IgG antibody (1 mg/mL) (Cat. No. I-2000-1; Vector Laboratories Inc., Newark, CA, USA) was used.

It is important to consider that αENaC is a continuous variable, meaning that it is expressed in platelets from both healthy and hypertensive individuals. Therefore, the test was designed to provide information regarding antigen concentration, given that high αENaC expression (the biomarker) is directly proportional to antigen levels in the sample and, consequently, to hypertension status. To achieve this, the anti-αENaC antibody was dispensed onto the nitrocellulose membrane in the designated test zone using a herringbone pattern consisting of several points [20]. This modification replaced the conventional single test line of the LFIA with a structured array of three herringbone units, each composed of three aligned spots, resulting in a total of nine spots. This layout was intended to facilitate easier visual interpretation and semi-quantitative assessment of αENaC expression.

The control antibody was applied in two points on each membrane. After spotting, the membrane was allowed to dry for 1 h at 37 °C to ensure proper antibody immobilization.

### 2.8. Evaluation of the Minimum Concentration of Recombinant αENaC Detectable by a Lateral Flow Immunoassay

To determine the detection threshold of the lateral flow immunoassay (LFIA), varying concentrations of the recombinant human αENaC protein (amino acids 20–44) (Cat. No. PEP-088, Invitrogen) were tested: 0.1, 0.25, and 0.5 μg/μL. These protein samples were functionalized with gold nanoparticles (AuNPs) and applied to the assay. For signal detection, the anti-αENaC antibody was dispensed onto the nitrocellulose membrane in a herringbone pattern consisting of nine discrete deposition points in the test zone. Additionally, two spots were used for control antibody application on each membrane. After antibody spotting, the strips were dried at 37 °C for 1 h. The optimal concentration was defined as the lowest protein concentration producing a strong and specific signal without background noise or nonspecific binding.

### 2.9. Blood Sample Collection

Venous blood samples were collected from participants at the Unidad de Medicina Familiar No. 44 of the Instituto Mexicano del Seguro Social (UMF 44, IMSS) in Mexico City. A total of 200 individuals, aged 16 to 80 years, were enrolled in this double-blind study. Blood was drawn into EDTA-containing vacuum tubes (Cat. No. P130754mL-K2, Wego, Weihai, China) to prevent coagulation. The study cohort included both individuals with a confirmed diagnosis of hypertension and those without a known clinical history of the condition at the time of sample collection. Table 1 presents the anthropometric and demographic characteristics of the participants. Exclusion criteria were pregnancy, withdrawal of informed consent, and visibly hemolyzed blood samples.

### 2.10. Platelet Isolation

Blood samples were centrifuged at 1300 rpm for 14 min, and the resulting platelet-rich plasma was mixed with an equal volume of adenosine (0.5 mmol/L, Cat. No. A9251, Sigma-Aldrich Co., St. Louis, MO, USA) and centrifuged again at 1300 rpm for 8 min. The resulting pellet was washed with 1× PBS and resuspended in the same buffer. Platelets were lysed in 50 μL of the lysis buffer (RIPA Buffer pH 8: 5 M NaCl, Tris pH 8, 100% Triton X-100, 50× protease inhibitor cocktail, and 10% SDS) and stored at −20 °C until analysis.

### 2.11. Determination of ENaC Concentration in Platelet Lysates

The total protein concentration in platelet lysates was quantified using the bicinchoninic acid (BCA) assay [21]. Absorbance measurements were performed using a NanoDrop 1000 spectrophotometer (NanoDrop, Thermo Scientific, USA). To determine the minimum protein concentration needed for specific ENaC detection in the immunoassay, protein concentrations of 0.5, 1.0, 1.5, and 2.0 μg/μL (in 10 μL aliquots) were tested. A concentration of 1.5 μg/μL was selected as the optimal level, balancing detection sensitivity and the minimization of nonspecific signals.

### 2.12. Detection of the ENaC in Platelet Lysates Using the Lateral Flow Immunoassay (LFIA)

Each of the 200 platelet lysates, adjusted to 1.5 μg/μL of the total protein, was applied (10 μL) to the sample pad of the LFIA strip, followed by 85 μL of the running buffer. The strips were allowed to develop for 15 min. The proper function of each strip was confirmed by the appearance of the control line signal.

### 2.13. Western Blotting

Parallel validation was performed using Western blot analysis. Platelet lysates were mixed with the loading buffer (Tris-HCl-SDS pH 6.8, glycerol, SDS, β-mercaptoethanol, and bromophenol blue), heated at 100 °C for 5 min, and subjected to SDS-PAGE using 12% polyacrylamide gels at 120 V. Proteins were transferred to nitrocellulose membranes (Hybond-Nb, Amersham Pharmacia, GE Healthcare, Buckinghamshire, UK) at 0.3 A for 70 min.

Membranes were blocked with 15% non-fat dry milk (Svelty) in TBST (25 mM Tris, 140 mM NaCl, 3 mM KCl, and 0.05% Tween-20) for 1 h and incubated overnight at 4 °C with primary antibodies: anti-ENaC (1:100; Cat. No. PA1-920A; Invitrogen, USA) and anti-GAPDH (1:1000; Cat. No. Sc-365062; Santa Cruz Biotechnology, USA) as a loading control.

Following three 10 min TBST washes, membranes were incubated for 1 h at room temperature with HRP-conjugated anti-rabbit and/or anti-mouse secondary antibodies (1:4000; Cat. No. 111-035-003; Jackson ImmunoResearch, West Grove, PA, USA). The excess antibody was removed with three TBST washes, and bands were visualized using enhanced chemiluminescence (ECL; Millipore, Burlington, MA, USA) and developed on an X-ray film (Kodak, Rochester, NY, USA).

### 2.14. Blinding and Statistical Methods

One investigator was responsible for attending the clinic, enrolling participants, and coding the samples. All assays and data analyses were carried out independently by other investigators who were blinded to the sample identities. The samples were only decoded after data analysis was completed. Statistical analyses were performed using the GraphPad Prism 10 software (La Jolla, CA, USA). All *p*-values reported are two-sided, with values of *p* < 0.05 considered statistically significant.

## 3. Results

### 3.1. Characteristics of AuNPs

**Figure 2:**
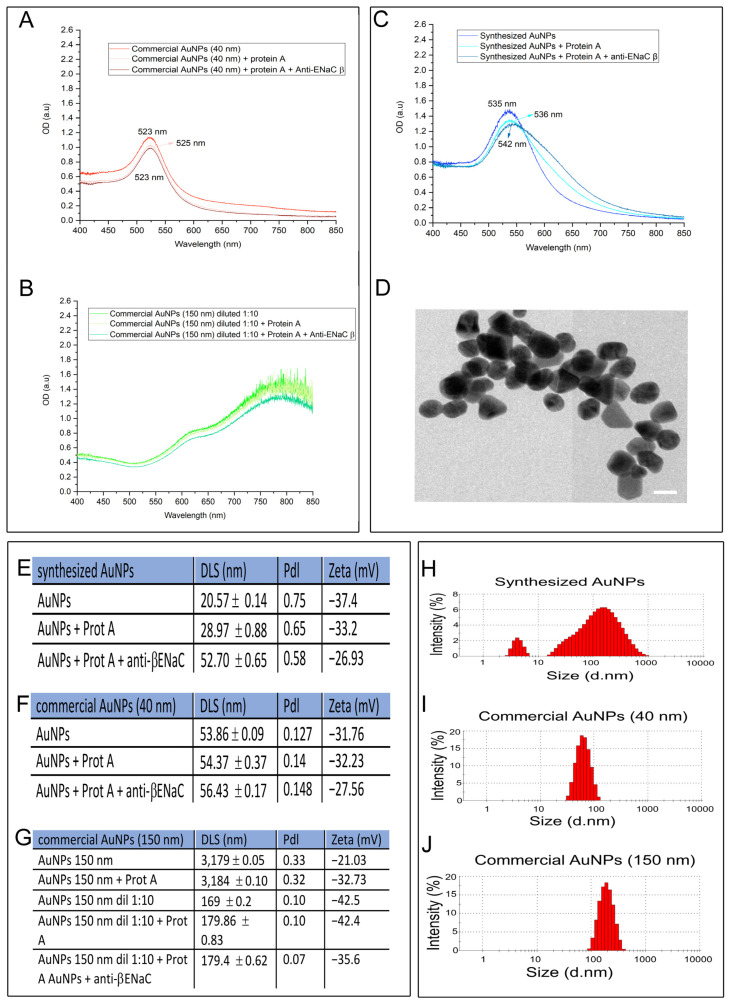
Characterization of gold nanoparticles (AuNPs). (**A**). UV-Vis absorption spectra of 40 nm AuNPs. Spectral comparison of commercial 40 nm AuNPs before and after surface functionalization with Protein A and the anti-βENaC antibody, highlighting characteristic plasmon resonance shifts indicative of successful conjugation. (**B**). UV-Vis spectra of 150 nm AuNPs (1:10 dilution). The optical response of larger AuNPs and the corresponding spectral shift following functionalization were used to evaluate conjugation efficiency and size-related optical behavior. (**C**). UV-Vis spectra of Synthesized ~30 nm AuNPs. Comparison between native and functionalized lab synthesized AuNPs, showing changes in surface plasmon resonance as evidence of successful bioconjugation. (**D**). Transmission electron microscopy (TEM) of native AuNPs TEM image showing the morphology and heterogeneity of the synthesized AuNPs prior to functionalization. Variability in shape is visible. Scale bar = 25 µm. (**E**). Summary table for synthesized AuNPs. Dynamic light scattering (DLS)-based measurements of the hydrodynamic diameter (d.nm), polydispersity index (PDI), and zeta potential (mV) for lab-synthesized AuNPs after functionalization. (**F**). Summary table for 40 nm commercial AuNPs. DLS, PDI, and zeta potential values for commercially available 40 nm AuNPs post-functionalization. (**G**). Summary table for 150 nm commercial AuNPs. Corresponding measurements for functionalized 150 nm AuNPs, showing size and dispersion characteristics. (**H**). Size distribution of functionalized synthesized AuNPs. Graph of particle size (nm) versus intensity (%) showing the distribution profile and homogeneity of synthesized AuNPs after bioconjugation. (**I**). Size distribution of functionalized 40 nm AuNP intensity (%) versus size (nm) graph for commercial 40 nm AuNPs after surface functionalization, indicating particle stability. (**J**). Size distribution of functionalized 150 nm AuNPs. Graph illustrating the particle size distribution of functionalized 150 nm AuNPs, highlighting uniformity and aggregation status.

The initial step in optimizing the lateral flow immunoassay (LFIA) was to determine the most suitable size of gold nanoparticles (AuNPs). Three approximate nanoparticle sizes (30, 40, and 150 nm) were evaluated using both commercially available and laboratory-synthesized AuNPs (Figure 2). To enhance assay specificity and stability, we opted to functionalize AuNPs with Protein A, which binds the Fc region of antibodies, facilitating optimal antibody orientation.

The minimum concentration of Protein A required to stabilize the AuNPs was determined following the protocol described in 2013 [19]. Various concentrations of Protein A (0.2, 0.3, 0.5, 1.0, 3.0, 6.0, and 9.0 μg/mL) were tested. A concentration of 3 μg/mL was identified as the lowest concentration that maintained nanoparticle stability.

Subsequently, the AuNP–Protein A complex was incubated for 1 h with the anti-β-ENaC monoclonal antibody at a concentration of 0.5 μg/mL, which was previously determined to be the optimal concentration. The functionalization of AuNPs with Protein A and anti-βENaC was confirmed through UV–visible spectroscopy, dynamic light scattering (DLS), zeta potential analysis, and transmission electron microscopy (TEM).

Among the options evaluated, synthesized AuNPs were considered most suitable for LFIA development. As shown in Figure 2C, a shift in the λ_max (surface plasmon resonance peak) after functionalization confirmed surface modification of the nanoparticles. In contrast, no significant shift in λ_max was observed for the commercial 40 nm AuNPs, although their narrower peak suggested greater uniformity in size distribution (Figure 2A). The 150 nm AuNPs produced overly concentrated suspensions that hindered accurate absorbance measurements, even after dilution, and their colorimetric performance was suboptimal (Figure 2B). TEM images confirmed the size and polydispersity of the AuNPs (Figure 2D).

Dynamic light scattering (DLS) measurements confirmed the selection of the synthesized AuNPs, as these demonstrated an increase in hydrodynamic diameter following functionalization. Specifically, the hydrodynamic radii increased from 20.57 ± 0.14 nm (bare AuNPs) to 28.97 ± 0.88 nm (AuNPs + Protein A) and to 52.70 ± 0.65 nm (AuNPs + Protein A + anti-βENaC) (Figure 2E). In contrast, commercial 40 nm AuNPs showed only minor changes in size: 53.86 ± 0.09 nm (bare), 54.37 ± 0.37 nm (with Protein A), and 56.43 ± 0.17 nm (with Protein A + anti-βENaC) (Figure 2F).

Measurements for commercial 150 nm AuNPs yielded significantly higher values: 3179 ± 0.05 nm (bare) and 3184 ± 0.10 nm (AuNPs + Protein A). Even after dilution, these particles failed to produce interpretable DLS data, with values of 169 ± 0.2 nm (diluted bare), 179.86 ± 0.83 nm (diluted + Protein A), and 179.4 ± 0.62 nm (diluted + Protein A + anti-βENaC) (Figure 2G). The distribution of DLS measurements is shown graphically in Figure 2H–J.

Synthesized AuNPs exhibited the greatest size variability, as reflected in their polydispersity index (PdI), which was consistent with prior characterizations (Figure 2E–G). These results support the use of synthesized AuNPs for the lateral flow immunoassay (LFIA), given their consistent and observable size changes upon functionalization.

Zeta potential analysis indicated values far from zero in all cases, confirming nanoparticle stability. Synthesized AuNPs displayed the highest absolute zeta potential, correlating with their experimentally observed stability and prolonged colloidal suspension without precipitation. Zeta potential values decreased after functionalization with Protein A and anti-β-ENaC across all nanoparticle types, ranging from −26.9 mV to −42.5 mV (Figure 2E–G).

### 3.2. Optimization of Lateral Flow Immunoassay (LFIA) Components

To optimize LFIA performance, several components were evaluated:

Running Buffer: Among the different formulations tested, the buffer composed of 0.01 M PBS, 1% Tween-20, and 1% BSA provided the most consistent and efficient flow across the test strip. As shown in Supplemental Figure S1, this buffer supported homogeneous nanoparticle migration to the excess pad without aggregation or signal loss.

Nitrocellulose Membrane Porosity: Two membrane types were evaluated—a 50 μm pore size with a flow time of 180 s/4 cm and a 100 μm pore size with a flow time of 75 s/4 cm (Hi-Flow™ Plus, MERCK). Supplemental Figure S2 shows that the 100 μm, 75 s/4 cm membrane offered better flow uniformity and speed and was therefore selected for the final assay design.

### 3.3. Calibration Curve and Limit of Detection for ENaC

To determine the minimal detectable concentration of the epithelial sodium channel (ENaC) using the lateral flow immunoassay (LFIA), we employed recombinant ENaC protein and applied the three-sigma threshold rule (three standard deviations from the mean) [22].

**Figure 3:**
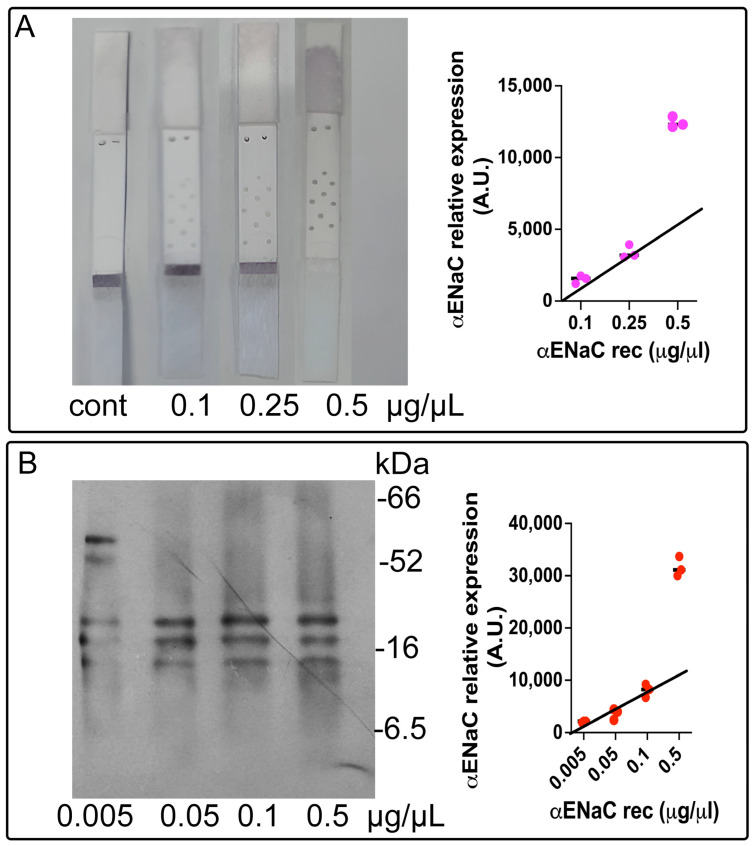
Calibration curves for recombinant ENaC detection using the LFIA and western blot. (**A**). LFIA-based calibration curve for ENaC. Dose–response curve obtained from the lateral flow immunoassay (LFIA) using increasing concentrations of the commercial recombinant ENaC protein. The test line intensity correlates with antigen concentration, confirming the assay’s sensitivity and semi-quantitative capability. (**B**). Western blot-based calibration curve for ENaC. Quantification curve of band intensity from Western blot analysis using the same concentrations of the recombinant ENaC as in the LFIA, serving as a comparative reference method. Data are presented as the mean ± SEM of at least three independent experiments.

A calibration curve was established using the commercially available purified ENaC protein across a range of concentrations (0.1, 0.25, and 0.5 µg/mL). The LFIA signal response was consistent and linear for the concentrations of 0.1 and 0.25 µg/mL; however, the signal corresponding to 0.5 µg/mL fell outside the linear range (Figure 3A). The limit of detection (LOD) for the ENaC using the LFIA was calculated to be 0.072 µg/µL, based on the 3σ rule.

Western blotting (WB) was employed as the reference method, given its established sensitivity and specificity. A calibration curve (Figure 3B) was generated using ENaC concentrations of 0.005, 0.05, 0.1, and 0.5 µg/mL. A strong linear correlation was observed in the range of 0.005 to 0.1 µg/mL. The LOD for the ENaC by WB was calculated as 0.077 µg/µL, also following the 3σ rule.

These results demonstrate that both the LFIA and WB exhibit high sensitivity, validating the LFIA as a reliable alternative for ENaC quantification in biological samples.

### 3.4. Clinical Classification and Anthropometric Characteristics of Participants

All participants in the study completed a brief survey and had their blood pressure measured at the time of blood collection using a validated oscillometric semi-automatic sphygmomanometer. Based on these measurements, the 200 individuals were classified into two groups: normotensive individuals (NTI, *n* = 85) and patients with hypertension (HTN, *n* = 115). Classification was based on the 2017 American College of Cardiology/American Heart Association (ACC/AHA) Hypertension Guidelines [5], where normal blood pressure is defined as systolic/diastolic < 120/80 mm Hg, elevated blood pressure as 120–129/<80 mm Hg, and hypertension (HTN) as ≥140/90 mm Hg.

The participants’ ages ranged from 25 to 90 years, with the HTN group being significantly older. The median age for the NTI group was 53.1 years, compared to 63.8 years for the HTN group (*p* < 0.0001). The sex distribution between the NTI and HTN groups was comparable (*p* = 0.710).

Patients in the HTN group had significantly higher median systolic blood pressure values (142.7 mm Hg) compared to the NTI group (127.9 mm Hg, *p* < 0.0001). Similarly, the median diastolic pressure was higher in the HTN group (90.15 mm Hg) than in the NTI group (78.78 mm Hg, *p* < 0.0001) (Table 1).

Potential confounding comorbidities such as diabetes mellitus, dyslipidemia, and obesity were analyzed but did not show statistically significant differences between groups. Diabetes was the most prevalent comorbidity in both groups (36.5% in NTI vs. 44.4% in HTN; *p* = 0.300). Interestingly, dyslipidemia was more frequent in the NTI group (15.3%) than in the HTN group (7.8%; *p* = 0.421). The mean BMI was similar between groups: 28.6 kg/m^2^ in NTI and 30.4 kg/m^2^ in HTN.

Lifestyle habits such as alcohol and cigarette consumption were also considered, given their potential contribution to hypertension development. Cigarette use was similar between the HTN and NTI groups (14.1% vs. 15.7%; *p* = 0.760). However, alcohol consumption was significantly higher in the HTN group compared to NTI (34% vs. 10.5%; *p* = 0.02). Interestingly, a higher proportion of HTN individuals reported engaging in physical activity (24.5%) compared to the NTI group (9%; *p* = 0.05).

Antihypertensive medication use was exclusive to individuals with a prior HTN diagnosis (Table 2). Among them, 46.3% (*n* = 50) were treated with combination therapy, including angiotensin-converting enzyme inhibitors (ACEIs) combined with beta blockers (BBs) or calcium channel blockers (CCBs), or angiotensin receptor blockers (ARBs) combined with BBs, CCBs, or diuretics. Monotherapy regimens included ARBs in 27 patients (25%), ACEIs in 16 patients (14.8%), BBs in 3 patients (2.8%), and CCBs in 7 patients (6.5%). Additionally, three patients (2.8%) reported managing their hypertension through their diet, and two patients (1.8%) used diuretics alone (Table S1).

To assess the diagnostic performance of the developed test, hypertensive subjects (*n* = 115) and normotensive individuals (*n* = 85) were classified based on their arterial tension values. The discriminatory capacity of the assay was evaluated using the area under the receiver operating characteristic (ROC) curve, which was generated with GraphPad Prism (La Jolla, CA, USA). A cut-off value (c) was established, above which measurements were indicative of hypertension. From this, the sensitivity and specificity of the test were determined.

A double-blind methodology was employed to validate the optimized lateral flow immunoassay (LFIA) by comparing its performance with the reference standard Western blotting. All samples were analyzed in parallel using both techniques. To standardize the protein concentration across samples, several dilutions of the total platelet protein (containing ENaC) were tested. Based on initial evaluations using a 0.5 μg/μL concentration, a final concentration of 1.5 μg/μL was selected as it enabled clearer differentiation between normotensive (NT) and hypertensive (HTN) individuals (Supplemental Figure S3).

**Figure 4:**
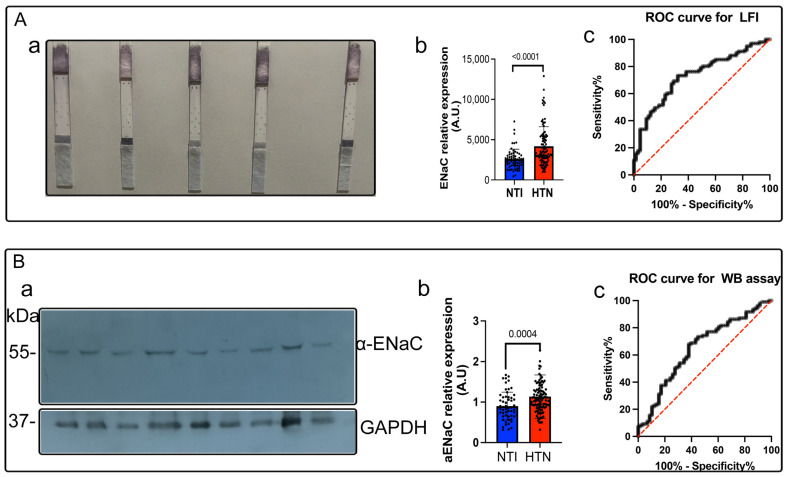
Detection of epithelial sodium channel (ENaC) expression in platelet plasma membranes. (**A**). Lateral flow immunoassay (LFIA): (**Aa**). Representative LFIA results from platelet lysates collected from individuals regardless of hypertension status, showing visible spot intensity corresponding to ENaC expression. (**Ab**). Quantitative analysis of ENaC expression in platelet membranes, represented by relative pixel intensity values. Statistical significance: *p* < 0.0001. (**Ac**). Receiver operating characteristic (ROC) curve generated from LFIA data, illustrating the assay’s diagnostic performance (*p* < 0.0001). (**B**). Western blotting (WB): (**Ba**). Representative immunoblots showing the α-ENaC protein (55 kDa) and GAPDH (37 kDa) as a loading control in platelet lysates. (**Bb**). Densitometric quantification of α-ENaC expression based on band intensity, expressed as relative pixel values. Statistical significance: *p* = 0.0004. (**Bc**). ROC curve generated from Western blot data to assess diagnostic performance (*p* < 0.0004). Data are presented as the mean ± SEM from at least three independent experiments.

Figure 4A displays representative LFIA test results from five individuals. The optical density of the developed spots allowed for clear classification into the hypertensive and normotensive groups. Notably, individuals with elevated blood pressure who had not been previously diagnosed were readily identified based on their elevated α-ENaC expression levels using the LFIA and were consequently reclassified, excluding them from the normotensive group (Figure 4).

Figure 4B presents representative 55 kDa bands corresponding to α-ENaC from nine different individuals, along with GAPDH bands used as a loading control. Each lane was loaded with 50 µg/mL of the total platelet protein. Densitometry analysis revealed significantly higher α-ENaC expression in HTN samples compared to NTI (mean values: 1.28 vs. 0.76; *p* = 0.0004; Figure 4B).

Using the resulting data, receiver operating characteristic (ROC) curves were constructed to evaluate the discriminatory capacity of the ENaC as a biomarker across the LFIA (Figure 4A) and WB (Figure 4B). The LFIA yielded an area under the curve (AUC) of 0.7314, with a sensitivity of 76.24% (CI: 66.74–84.14%), a specificity of 61.54% (CI: 48.64–73.35%), and a cutoff value of 2660. In comparison, the Western blot showed an AUC of 0.6491, with 68.31% sensitivity (CI: 59.60–76.74%), 60.34% specificity (CI: 47.49–71.91%), and a cutoff value of 0.9066 (Table 2).

Elevated levels of the epithelial sodium channel (ENaC) were consistently observed in a subset of individuals within the study cohort, and this overexpression demonstrated a significant association with elevated blood pressure. As a biomarker, ENaC expression measured by the lateral flow immunoassay (LFIA) exhibited a sensitivity of 76.2% and a specificity of 61.5%, indicating its potential utility in detecting a substantial proportion of individuals at risk for hypertension.

**Figure 5:**
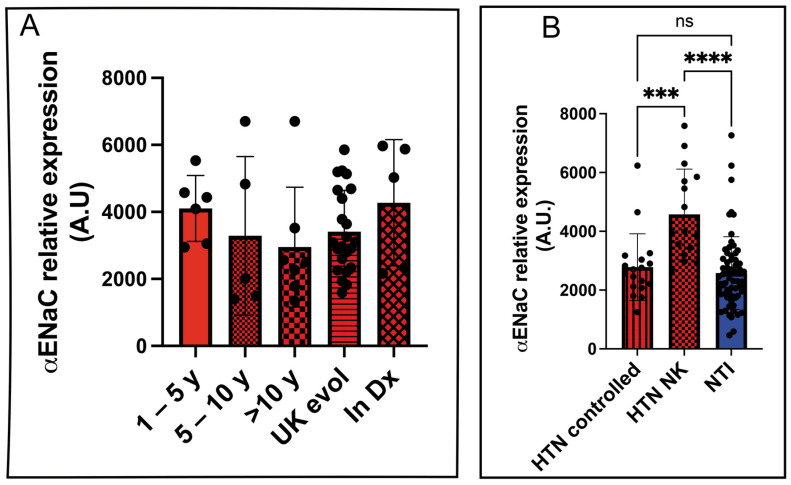
Diagnostic accuracy of the lateral flow immunoassay (LFIA) for detecting αENaC expression in hypertensive patients. (**A**). Relative expression levels of αENaC in platelet lysates from hypertensive individuals, categorized by disease duration: 1–5 years, 5–10 years, more than 10 years, unknown evolution time (UK evol), newly diagnosed (In Dx), controlled hypertension (HTN controlled), and undiagnosed hypertension (HTN NK). (**B**). Comparative analysis of αENaC expression levels among three groups: hypertensive patients with controlled blood pressure (HTN controlled), individuals with undiagnosed hypertension (HTN NK), and normotensive individuals (NTIs). Data are presented as the mean ± SEM from the LFIA quantification of spot intensity. Statistical significance was determined using appropriate tests as indicated in the main text. *** *p* = 0.0002; **** *p* < 0.0001.

To evaluate the potential of αENaC as a biomarker for disease progression, we analyzed its relative expression in platelet lysates from hypertensive patients stratified by the duration of their diagnosis: 1–5 years, 5–10 years, more than 10 years, unknown duration, and newly diagnosed. However, no statistically significant differences in αENaC expression were observed among these subgroups (Figure 5A).

We further compared αENaC expression levels between hypertensive patients with controlled blood pressure (HTN controlled) and those who were unaware of their condition (HTN NK). A statistically significant difference was found between these two groups (2782 ± 267.5 vs. 4574 ± 385.1; *p* = 0.0002). Similarly, the difference between HTN NK and normotensive individuals (NTIs) was also statistically significant (4574 ± 267.5 vs. 2582 ± 385.1; *p* < 0.0001), highlighting the diagnostic capability of the LFIA assay to detect undiagnosed hypertensive individuals based on elevated αENaC levels.

As expected, there was no statistically significant difference in αENaC expression between hypertensive patients with controlled blood pressure and normotensive individuals (2782 ± 267.5 vs. 2582 ± 153.2; *p* = 0.8247) (Figure 5B).

## 4. Discussion

High blood pressure is one of the leading risk factors for death and disability worldwide. Between 1990 and 2019, the number of people living with hypertension doubled from 650 million to 1.3 billion [23]. According to the World Health Organization (2023), among adults aged 30–79 years with hypertension, only 54% have been diagnosed, 42% are receiving treatment, and merely 21% have their condition under control.

The identification of reliable biomarkers for hypertension remains an area of active research, as no single biomarker is currently applicable for diagnosis or effective management in all patients [24]. We previously proposed that the elevated expression of the epithelial sodium channel (ENaC) in platelets could serve as a potential biomarker for hypertension diagnosis [11], and we later validated its clinical utility [14]. Seeking a more practical and accessible diagnostic approach, we developed a lateral flow immunochromatographic assay (LFIA)—to our knowledge, the first of its kind, —capable of detecting the ENaC biomarker in a significant number of cases. Notably, ENaC expression remains unaffected by oxidative stress or inflammatory conditions, making it a stable and reliable biomarker for hypertension screening.

To enhance specificity, we adopted a sandwich-format LFIA, which leverages double antigen–antibody recognition, making it ideal for complex biological samples [25]. For improved sensitivity, AuNPs were functionalized with Protein A, enabling proper orientation of the Fc region of the antibody and leaving the Fab region available for antigen binding [26]. We conjugated the nanoparticles with a monoclonal anti-α-ENaC antibody and used a polyclonal anti-β-ENaC antibody as the capture antibody on the membrane, ensuring multi-epitope recognition and efficient binding [27].

Characterization using UV-Vis spectroscopy and DLS confirmed that our synthesized AuNPs outperformed commercial ones, despite minor heterogeneity in size [14,28]. TEM imaging showed spherical nanoparticles measuring 30–35 nm, consistent with reports linking surface curvature to protein-binding efficiency [29,30].

The optimization of physical and chemical properties of the test membrane was critical. A pore size of 100 µm yielded optimal flow (75 s/4 cm), ensuring sufficient antigen–antibody interaction while maintaining rapid test usability.

We used herringbone patterns to enhance the visualization of the LFIA signal [20], allowing results to be interpreted without the need for sophisticated equipment and thereby facilitating detection with the naked eye. However, further optimization is necessary to fully achieve this objective. Additionally, it is important to note that the spots containing the AuNPs and anti-ENaC used to form the herringbone pattern, along with the control spots, were manually applied to the strips. This manual placement may introduce variability in signal intensity and reading accuracy due to potential inconsistencies in spot deposition. Therefore, a densitometric analysis approach was implemented, based on image capture and quantification [31].

The diagnostic performance of the lateral flow immunoassay (LFIA) was evaluated in a double-blind study using platelet lysates obtained from 200 individuals from an open population cohort. Receiver operating characteristic (ROC) curve analysis was conducted to assess the assay’s ability to discriminate between hypertensive and normotensive individuals, treating ENaC expression as a continuous variable. Additionally, patient characteristics and comorbidities—including diabetes and obesity—were taken into account during the analysis. No statistically significant differences were observed between the hypertensive and normotensive groups in relation to these variables, indicating that the diagnostic accuracy of the assay is independent of common metabolic comorbidities.

The LFIA yielded results comparable to Western blotting, the current gold standard. The area under the ROC curve (AUC) for the LFIA was 0.7314—approaching the clinical utility threshold of 0.75 [32,33]. Notably, the assay identified 44.82% of participants as hypertensive who were previously unaware of their condition, aligning with national reports for undiagnosed hypertension in Mexico [34].

Our optimized LFIA enabled the detection of elevated levels of the epithelial sodium channel (ENaC), a biomarker significantly associated with hypertension, with diagnostic performance comparable to that of Western blotting. While ENaC overexpression does not define a definitive diagnosis of hypertension, its detection proved valuable in identifying individuals with elevated blood pressure, including previously undiagnosed cases, and distinguishing between controlled and uncontrolled hypertensive profiles. These findings support the utility of the ENaC as a complementary biomarker in screening strategies aimed at early risk identification and improved clinical decision-making.

## 5. Conclusions

Based on the premise of utilizing the ENaC as the first specific biomarker for hypertension (HTN), we successfully optimized a lateral flow immunochromatographic assay (LFIA) targeting the overexpression of this channel in platelet membranes of hypertensive individuals. A strong correlation was observed between the intensity of the colored spots and the ENaC concentration, reinforcing the assay’s potential as a valuable tool for biomarker detection. A double-blind clinical trial further validated the utility of the assay, confirming its effectiveness in distinguishing ENaC expression in a significant number of hypertensive patients compared to normotensive individuals.

Although the optimization of the sandwich immunoassay produced promising results, several technical limitations were encountered during its development and implementation, which must be addressed to enhance accuracy, reproducibility, and overall performance. Despite these challenges, the findings lay a solid foundation for the future integration of this diagnostic tool into healthcare systems, with the aim of supporting early hypertension detection and improving disease management.

## Figures and Tables

**Table 1:** Anthropometric characteristics of healthy individuals and patients with HTN.

	Normotensive Individuals (NTI) (*n* = 85)	Diagnosed Hypertensive (DHTN) (*n* = 115)	*p* Value
Median age	53.1	63.8	<0.0001
Women/men (%)	69.4/30.6	66.9/33.0	0.710
Systolic mmHg, mean	127.9	142.7	<0.0001
Diastolic mmHg, mean	78.78	90.15	<0.0001
Diabetic (%)	36.5	44.4	0.300
Dyslipidaemia (%)	15.3	7.8	0.421
BMI (kg/m^2^) mean ± SD	28.6	30.4	0.39
Number of current smokers (%)	14.1	15.7	0.760
Alcohol consumers (%)	10.5	34	0.02
Exercise practitioners (%)	9	24.5	0.05

**Table 2:** ROC parameters for the Wester blotting assay and the LFI assay performed to evaluate the expression of the ENaC on platelets.

Assay	Cutoff	Area Under the ROC Curve	Sensitivity (CI)	Specificity (CI)
AuNPs-antiENaC	2660	0.7314	76.24% (0.6674–0.8414)	61.54% (0.4864–0.7335)
Western blotting	0.9066	0.6491	68.31% 0.5960–0.7674)	60.34% (0.4749–0.7191)

## Data Availability

The original contributions presented in this study are included in the article. Further inquiries can be directed to the corresponding author(s).

## Supplementary Materials

The following supporting information can be downloaded at https://www.mdpi.com/article/10.3390/bios15070399/s1, Figure S1. Evaluation of run buffers for LFIA optimization. Figure S2. Evaluation of nitrocellulose membrane porosity and the flow rate for LFIA optimization. Figure S3. Determination of the minimum detectable concentration of αENaC by the LFIA. Table S1. Distribution of antihypertensive medication use among study participants.

## Abbreviations

The following abbreviations are used in this manuscript:

Abbreviation	Full Term
HTN	Hypertension
NTI	Normotensive Individual
LFIA	Lateral Flow Immunoassay
AuNPs	Gold Nanoparticles
ENaC	Epithelial Sodium Channel
WB	Western Blot
AUC	Area Under the Curve
ROC	Receiver Operating Characteristic
GAPDH	Glyceraldehyde 3-Phosphate Dehydrogenase
DLS	Dynamic Light Scattering
Pdl	Polydispersity Index
LOD	Limit of Detection
In Dx	In Diagnostic
HTN NK	Hypertensive Not Known
HTN controlled	Hypertension Controlled
UK evol	Unknown Evolution
