# Supplementary material for: ENaC Biomarker Detection in Platelets Using a Lateral Flow Immunoassay: A Clinical Validation Study
Source: Biosensors (Basel). 2025 Jun 20;15(7):399. doi: 10.3390/bios15070399 (PMC12292876; doi:10.3390/bios15070399)
Supplement: Supplementary file 1 [file biosensors-15-00399-s001.zip › biosensors-3665934-supplementary.pdf]

Table S1. Distribution of antihypertensive medication use among study participants

Anti-hypertensive agent	Number (%)
ARB	27 (25)
ACEI	16 (14.8)
BB	3 (2.8)
CCB	7 (6.5)
Diet	3 (2.8)
Diuretic	2 (1.8)
Combination of anti-hypertensive medication	50 (46.3)

ACEI = angiotensin-converting enzyme inhibitors, ARB = angiotensin II receptor blockers, BB = beta blockers, CCB = calcium channel blockers.

Supplemental Figure S1

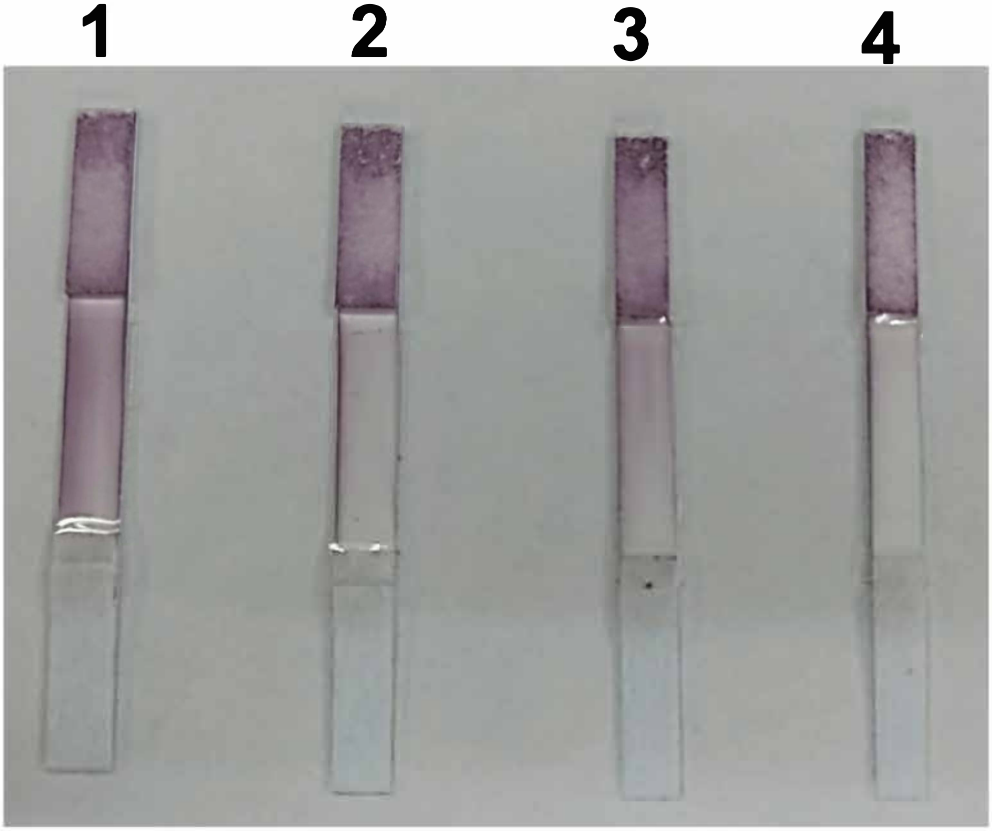

1. Borate Buffer
2. 1% BSA, 1% saccharose, 0.05M NaCl,
   0.05M L-arginine, 0.02%, 0.5% PEG plyvinil alcohol,
   0.2%Tween 20
3. Tris EDTA
4. 0.01 M PBS, 1% Tween 20, 1% BSA

Figure S1. Evaluation of run buffers for LFIA optimization.
LFIA strips were assembled and tested with different running buffers to determine optimal flow performance. A total volume of 85 μL of each buffer was applied to the sample pad. After 15 minutes, flow characteristics and nanoparticle homogeneity were evaluated visually. n = 5.

Supplemental Figure S2

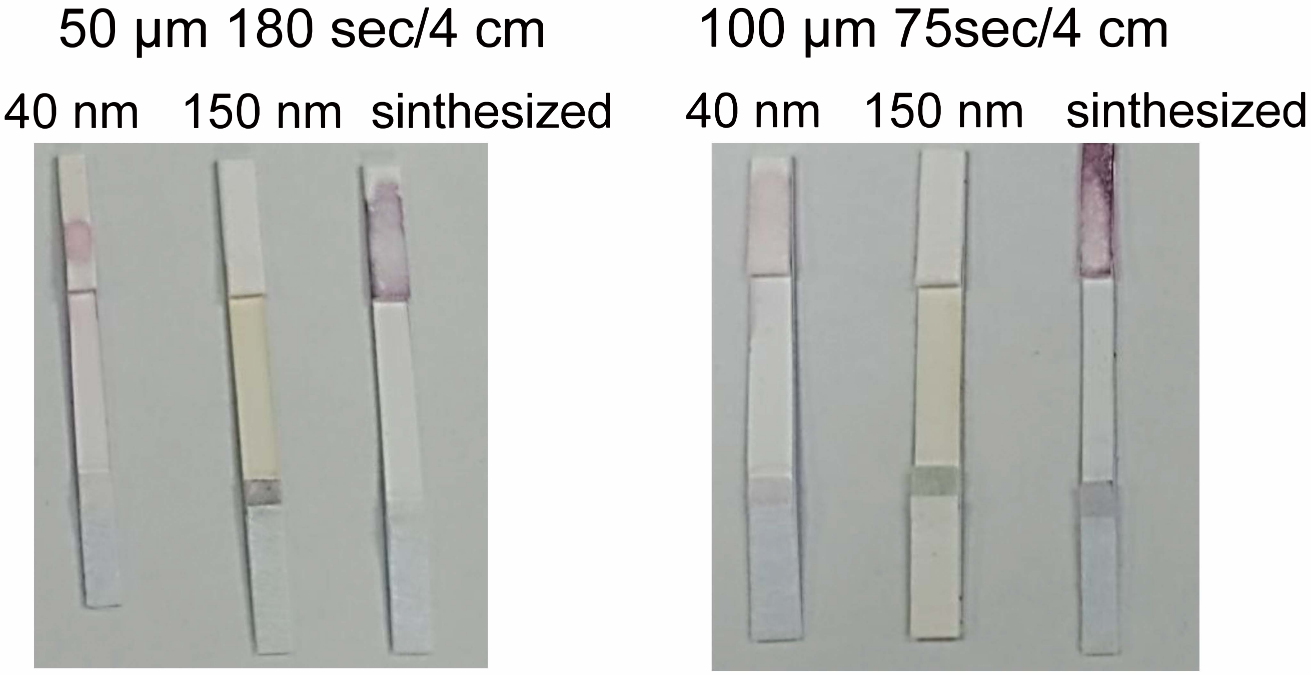

Figure S2. Evaluation of nitrocellulose membrane porosity and flow rate for LFIA optimization. Strips were assembled using membranes of varying porosity. Each was tested with 85 μL of a running buffer applied to the sample pad and observed for 15 minutes to assess flow rate and consistency. n = 5.

Supplemental Figure S3

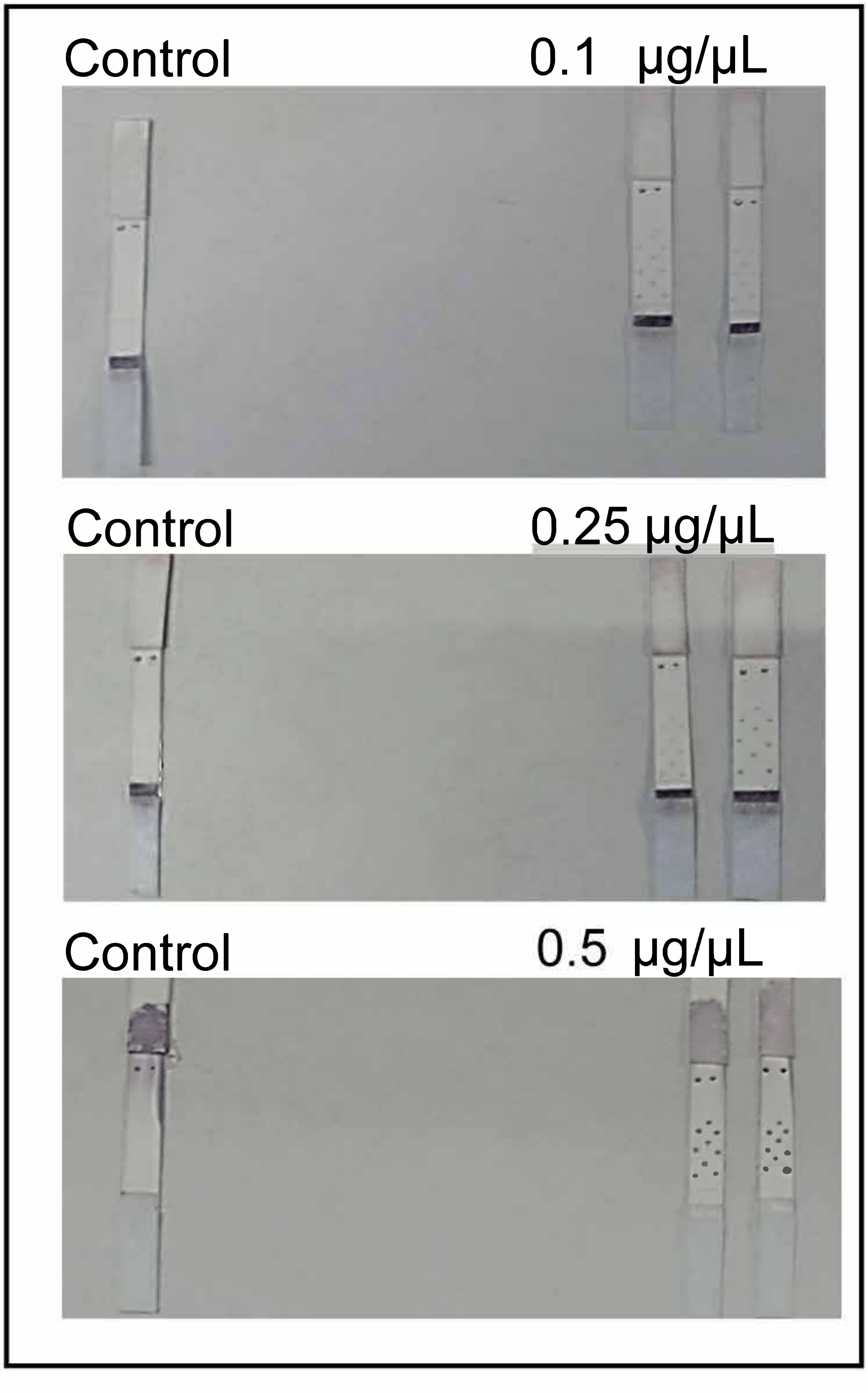

Figure S3. Determination of the minimum detectable concentration of αENaC by LFIA. Left panel. Control LFIA strips with only functionalized AuNPs and anti-IgG antibody at varying αENaC concentrations. Right panel. Strips additionally spotted with αENaC antibody in a herringbone pattern. Minimum effective detection observed at an αENaC antibody concentration of 0.2 *µ*g/mL.
